# Risk Factors for Development of Chronic Kidney Disease in Cats

**DOI:** 10.1111/jvim.13917

**Published:** 2016-03-06

**Authors:** N.C. Finch, H.M. Syme, J. Elliott

**Affiliations:** ^1^ Bristol RenalSchool of Clinical SciencesUniversity of BristolBristolUK; ^2^ Department of Clinical Science and ServicesRoyal Veterinary CollegeHatfieldHertsUK; ^3^ Department of Comparative Biomedical SciencesRoyal Veterinary CollegeLondonUK

**Keywords:** Feline, Predictors, Renal

## Abstract

**Background:**

Identification of risk factors for development of chronic kidney disease (CKD) in cats may aid in its earlier detection.

**Hypothesis/objectives:**

Evaluation of clinical and questionnaire data will identify risk factors for development of azotemic CKD in cats.

**Animals:**

One hundred and forty‐eight client‐owned geriatric (>9 years) cats.

**Methods:**

Cats were recruited into the study and followed longitudinally for a variable time. Owners were asked to complete a questionnaire regarding their pet at enrollment. Additional data regarding dental disease were obtained when available by development of a dental categorization system. Variables were explored in univariable and multivariable Cox regression models.

**Results:**

In the final multivariable Cox regression model, annual/frequent vaccination (*P* value, .003; hazard ratio, 5.68; 95% confidence interval, 1.83–17.64), moderate dental disease (*P* value, .008; hazard ratio, 13.83; 95% confidence interval, 2.01–94.99), and severe dental disease (*P* value, .001; hazard ratio, 35.35; 95% confidence interval, 4.31–289.73) predicted development of azotemic CKD.

**Conclusion:**

Our study suggests independent associations between both vaccination frequency and severity of dental disease and development of CKD. Further studies to explore the pathophysiological mechanism of renal injury for these risk factors are warranted.

**Abbreviations**
BAH: Beaumont Sainsbury Animals' Hospital
CKD: chronic kidney disease
CRFK: Crandell‐Rees feline kidney
DSH: domestic short hair
DLH: domestic long hair
FIV: feline immunodeficiency virus
GIS: geospatial information system
GFR: glomerular filtration rate
LTF: lost to follow‐up
KEEP: National Kidney Foundation's Kidney Early Evaluation Program
PDSA: People's Dispensary for Sick Animals
USG: urine specific gravity

Chronic kidney disease (CKD) has a high prevalence in both humans and domestic cats. In human patients across Europe, Asia, and North America the prevalence is reported to be 2.5–11.2%.^1^ The figure is similar in cats with approximately 10% of cats >10 years of age reported to be affected although the etiopathogenesis may have some differences.^2^ Chronic kidney disease is defined as a sustained decrease in renal function over at least 3 months. It is not a single entity but a heterogeneous syndrome resulting in loss of functioning renal mass. In veterinary patients, congenital or acquired disorders can lead to development of CKD. Acute kidney damage (single or repeated episodes) secondary to urinary obstruction, nephrotoxins, pyelonephritis, or ischemic injury also can progress to CKD. The disease syndrome is an important cause of morbidity and mortality in cats and as a result, there is much interest in identifying risk factors for disease. These risk factors may not only aid in early detection, but also may help in understanding disease pathogenesis and development of new treatments.

Few studies have explored risk factors and predictors of CKD in cats. Biochemical variables that predict development of azotemic CKD within 12 months have been reported.^3^ A recent retrospective study reported an increased odds of diagnosis of CKD in cats with weight loss, thin body condition, dehydration, a recent history of general anesthesia, prior diagnosis of periodontal disease or cystitis and being male.^4^ A case–control study reported hyperthyroidism, lower urinary tract disease, frequent vomiting, altered appetite, and weight loss to be risk factors for CKD in cats.^5^ However, many of these risk factors may have resulted from CKD rather than leading to its development. An earlier case–control study identified ad libitum feeding, low dietary fiber, various nutritional factors, and low body weight to be associated with increased odds of CKD.^6^ Other studies have suggested certain breeds of cat, particularly the Maine coon, Abyssinian, Siamese, Russian blue, and Burmese^2^ and also cats infected with feline immunodeficiency virus (FIV)^7^ to have increased risk of CKD compared to control cats. Certain breeds such as the Birman normally may have higher serum creatinine concentrations which could lead to misclassification in these studies.^8^ Results of these previous studies should be interpreted with caution because they were of case–control design, having potential to introduce bias and confounders.^9^ A further limitation was collection of data retrospectively, which can introduce additional bias.

Dietary factors have been suggested to contribute to development of CKD in cats.^10^, ^11^, ^12^, ^13^ Feeding a commercially available high protein, acidifying diet which was potassium depleted led to hypokalemia and development of CKD in healthy cats, although the formulation of this diet is unlikely to represent typical commercial diets fed to cats.^10^ An earlier study found cats fed an acidifying diet developed both hypokalemia and azotemia, but was limited by the low number of cats included.^11^

Previous studies found cats given commercially available vaccines by SC injection developed anti‐Crandell‐Rees feline kidney (CRFK) proteins and antirenal tissue antibodies.^14^, ^15^ Furthermore, interstitial nephritis was identified in renal biopsy tissue collected after SC vaccination.^16^ A large proportion of the feline population receives regular vaccinations and, based on findings from previous studies described above, this practice may be considered a potential risk factor for CKD in cats. No epidemiological studies have evaluated the effects of vaccination on kidney function in cats.

Identification of risk factors and predictors for development of azotemia in cats would allow targeted population screening and identification of high‐risk patients. The information also would be important because it may provide an opportunity for early detection of the underlying disease and intervention to manage the primary disease process and prevent further renal damage. The aim of our study was to identify lifestyle and healthcare variables that predicted development of azotemia using a longitudinal cohort study.

## Materials and Methods

### Study population

Healthy geriatric (>9 years old) nonazotemic (plasma creatinine concentration <2.0 mg/dL [177 μmol/L]) cats were recruited into a longitudinal study between 2005 and 2009 that was conducted at 2 London‐based first opinion practices (Beaumont Sainsbury Animals' Hospital [BAH], Royal Veterinary College, Camden and People's Dispensary for Sick Animals [PDSA], Bow). Healthy cats were defined based on normal clinical history, physical examination and blood and urine screening. The study was conducted with approval from the Royal Veterinary College's Ethics and Welfare committee. Cats were followed longitudinally until either they developed azotemia, were lost to follow‐up (LTF), died, or the study end point (31.12.09) was reached. Reassessment of cats was performed at approximately 6‐month intervals in normal healthy cats. More frequent evaluations were performed in some cats depending on the clinical management required. At least 2 visits (1 to include the enrollment visit) were required for inclusion in the study. Cats that developed concurrent disease, such as hyperthyroidism, during the follow‐up period were censored. Data from cats that died or were LTF also were censored. At the initial and all subsequent visits, a full medical history was obtained and physical examination performed. At the study end‐point cats were categorized according to their renal status (azotemic or nonazotemic). Cats were classified as having azotemic CKD if they had a plasma creatinine concentration above the laboratory reference range (>2.0 mg/dL [177 μmol/L]) in association with decreased urine concentrating ability (USG < 1.035) or demonstrated persistently increased plasma creatinine concentration at 2 visits typically 6–8 weeks apart. Renal biopsies were not performed.

### Questionnaire data

At the initial visit, cat owners were asked to complete a questionnaire regarding their pet's lifestyle, dietary habits, and vaccination status. This questionnaire has been presented in a previous study evaluating risk factors for hyperthyroidism in cats.^17^ When questionnaires were not completed fully or further information was required owners were contacted retrospectively by telephone and additional information obtained. Information was collected regarding breed, sex, diet, environment, cigarette smoke exposure, vaccination, and history of dental disease. Because of the low numbers in each breed group, cats were classified as domestic short hair (DSH)/domestic long hair (DLH) or pedigree for statistical analysis. Dietary variables evaluated included type of food fed (wet versus dry) and feeding of urinary or senior diets. Information regarding the environment the cat lived in included whether the cat lived predominantly indoors or outdoors and whether the household was situated in an urban or semirural/rural environment. Vaccination status was categorized as (1) never vaccinated, (2) primary vaccination only, (3) occasional vaccination (>2‐year interval), (4) frequent or annual vaccination (every 1–2 years), or (5) unknown vaccination status. For the purposes of statistical analysis, never vaccinated, primary vaccination only and occasional vaccination categories were combined and renamed never/occasional vaccination. Data for dental disease included a dental disease category and history of previously diagnosed dental disease or treatment. Categories for variables are presented in Table 1.

**Table 1:** Results at baseline of the statistical analysis comparing cats remaining nonazotemic and cats developing azotemia

Variable		Nonazotemic cats (N = 121)	Azotemic Cats (N = 27)	*P* Value
Age (years)				.343
Median (range)		12.2 (9.0–21.8)	13.4 (9.9–20.1)	
Breed	**DSH/DLH**	104 (86%)	22 (81%)	.555
	Pedigree	17 (14%)	5 (19%)	
Sex	**FN**	71 (59%)	16 (59%)	.993
	MN	50 (41%)	11 (41%)	
Diet fed	**Predominantly dry food**	32 (26%)	9 (33%)	.345
	Approximately 50%:50%	30 (25%)	3 (11%)	
	Predominantly wet food	59 (49%)	14 (52%)	
Urinary diet fed	**No**	85 (70%)	20 (74%)	.562
	Yes	11 (9%)	1 (4%)	
	Unknown	25 (21%)	6 (22%)	
Senior diet fed	**No**	47 (39%)	9 (33%)	.595
	Yes	50 (41%)	14 (52%)	
	Unknown	24 (20%)	4 (15%)	
Environment	**Semirural/rural**	22 (18%)	3 (11%)	.413
	Urban	99 (82%)	24 (89%)	
Lifestyle	**Predominantly indoor**	86 (71%)	15 (56%)	.026
	Approximately 50%:50%	25 (21%)	4 (15%)	
	Predominantly outdoor	10 (8%)	8 (30%)	
Smoke exposure	**None/infrequent**	71 (59%)	13 (48%)	.383
	Frequent	44 (36%)	11 (41%)	
	Unknown	6 (5%)	3 (11%)	
Vaccination status	**Never/occasional vaccination**	78 (65%)	8 (30%)	.007
	Frequent/annual vaccination	26 (21%)	13 (48%)	
	Unknown	17 (14%)	6 (22%)	
Dental disease previously diagnosed or treated	**No**	58 (48%)	8 (30%)	.114
	Yes	38 (31%)	14 (52%)	
	Unknown	25 (21%)	5 (18%)	
Dental disease category	**No dental disease**	26 (21%)	2 (7%)	.001
	Mild	68 (56%)	9 (33%)	
	Moderate	15 (12%)	8 (30%)	
	Severe	11 (9%)	8 (30%)	

Categories in bold font are the reference categories against which other categories within the variable were compared in the Cox regression analysis.

A categorization system for dental disease was established and where possible applied to clinical findings recorded at initial evaluation. The veterinarian examining the cat awarded a calculus index of 0–3 and a gingivitis index of 0–3. These were combined and the sum of the index formed the dental disease category. Criteria for determining dental disease category are listed in Table 2. Where a gingivitis and calculus index was not recorded, dental disease category was defined based on oral examination findings described in the medical record by the examining clinician (61/148; 42%).

**Table 2:** Criteria for dental disease category

Dental Disease Category	Calculus Index Criteria	Calculus Index (0–3)	Gingivitis Index Criteria	Gingivitis Category (0–3)
No dental disease (0)	No calculus	0	No gingivitis	0
Mild (1–2)	Minimal layer of calculus visible on teeth at gingival margin	1	Thin area of mild inflammation at gingival margin	1
Moderate (3–4)	Moderate amount of calculus visible at gingival margin	2	Larger area of moderate inflammation affecting gingiva ± bleeding	2
Severe (5–6)	Large amount of calculus covering a significant surface area of the tooth and extending into interdental space	3	Severe inflammation of gingiva ± bleeding and stomatitis	3

Criteria for calculus and gingivitis index for assessing dental disease in cats at the initial evaluation. Calculus and gingivitis index were combined to give the dental disease category (no dental disease, mild, moderate, and severe).

### Geospatial information system (GIS) analysis of geographical data

Further analysis of environmental data was performed by evaluating geographical information. A GIS software program was employed that allowed epidemiological data to be analyzed and displayed spatially. Postcode data were obtained for cats included in the prospective study. The postcode of the address for which the cat had spent the predominant part of its life was used in the analysis. All cats for which data could not be accurately retrieved were excluded from analysis. Postcodes for the study area were downloaded from the UK Borders postcode directory (https://census.edina.ac.uk/pcluts.html) and the easting and northing co‐ordinates extracted for each cat based on postcode. Data were mapped in ArcMap9.3^a^ using a projected co‐ordinate system (British National Grid). The location of cats that remained nonazotemic and cats that developed azotemia was mapped.

Geographical clusters of azotemic cats were explored using saTScan8.0 software to determine the spatial scan statistic.^18^ This statistic was obtained by comparing the number of observed and expected cases occurring within the area of a randomly generated circle with the area outside the circle. The detection of clusters was performed using the Bernoulli probability model with a maximum cluster size of 50% of the total population (the default setting for the software). The *P* value of any clusters was obtained using Monte Carlo hypothesis testing where the number of simulations for Monte Carlo replications was 999.

### Statistical methods

Statistical analyses were performed using a statistical software package.^b^ Data were assessed for normality using the Kolmogorov–Smirnov test and by visual inspection of graphical plots. Descriptive statistics were performed to assess the distribution of data. Continuous variables were compared at baseline between cats that developed azotemia and cats that remained nonazotemic using the Mann–Whitney *U*‐test. Categorical variables were compared using Pearson's chi‐square test or Fisher's exact test. Where data were unknown or unavailable, the cat was placed into an unknown/missing data category to ensure results were not biased. Cox regression analysis was performed to identify variables that were independent predictors of development of azotemia. Data were censored if the cat developed concurrent disease, was LTF or died before the study end‐point. Univariable Cox regression analysis was performed initially. Variables with a *P* value < .2 in the univariable analysis subsequently were entered into a manual, forward selection, stepwise multivariable Cox regression model. Two‐way interactions of significant variables were explored by creating product terms. The product terms of any significant interactions were evaluated in the final multivariable model. The proportionality assumption was assessed by examining Kaplan–Meier and log‐minus‐log survival curves. Multicollinearity was assessed by evaluating the correlation matrix. Hazard ratios with 95% confidence intervals also were calculated. Kaplan–Meier curves were constructed for categorical variables significant in the final multivariable model to show the risk relationship for development of azotemia for each category. Statistical testing of Kaplan–Meier curves was performed using the log rank test. Significance was set at *P* < .05.

## Results

### Study population

Between January 2005 and December 2009, 343 healthy nonazotemic geriatric cats without evidence of concurrent disease were presented to the study group at 2 London‐based first opinion clinics. Of these, 148 (43%) cats did not return to the clinics for subsequent visits after the initial evaluation and therefore were excluded from the study. Of the 195 cats that were followed longitudinally over the study period, 39 (20%) developed concurrent nonrenal disease. These included hyperthyroidism (n = 36 [18%]), hepatic tumor (n = 1 [0.5%]), and hyperaldosteronism (n = 2 [1%]). Aldosterone concentrations were not routinely measured in all cats, rather the diagnosis of hyperaldosteronism was based on clinical suspicion and additional testing. Questionnaire data were available for analysis from 148 cats and was used for statistical analysis.

### Questionnaire data

Data from 148 cats were included in the final analysis of questionnaire data. Twenty‐seven (18%) cats developed azotemic CKD. At baseline, the median (range) plasma creatinine concentration, BUN concentration and USG in the cats that remained nonazotemic were 1.5 (0.6–2.0) mg/dL, 29.4 (16.8–62.7) mg/dL and 1.045 (1.013–1.090), respectively. The median (range) plasma creatinine concentration, BUN concentration and USG in the cats that developed azotemic CKD was 1.8 (1.2 –2.0) mg/dL, 37.2 (20.7–63.8) mg/dL and 1.032 (1.017–1.068), respectively. Plasma creatinine and BUN concentrations were significantly higher (*P* < .001) and USG significantly lower (*P* = .014) in cats that developed azotemic CKD. The median (range) number of days to development of azotemic CKD was 294 (14–1442). The median (range) number of days of follow‐up for cats remaining nonazotemic was 381 (14–1575). The mean incidence of development of azotemia within 12 months was 11%. The 22 pedigree cats included Persian (n = 7), Burmese (n = 5), Russian blue (n = 3), Bengal (n = 3), British short hair (n = 2), Balinese (n = 1), and British blue (n = 1).

The following variables were significantly different at baseline between cats remaining nonazotemic and cats developing azotemia: lifestyle (*P* = .026), vaccination status (*P* = .007) and dental disease category (*P* = .001). Results of statistical analyses comparing the 2 groups of cats using either the Mann–Whitney *U*‐test for continuous variables or chi‐square test for categorical variables are presented in Table 1.

**Figure 1:**
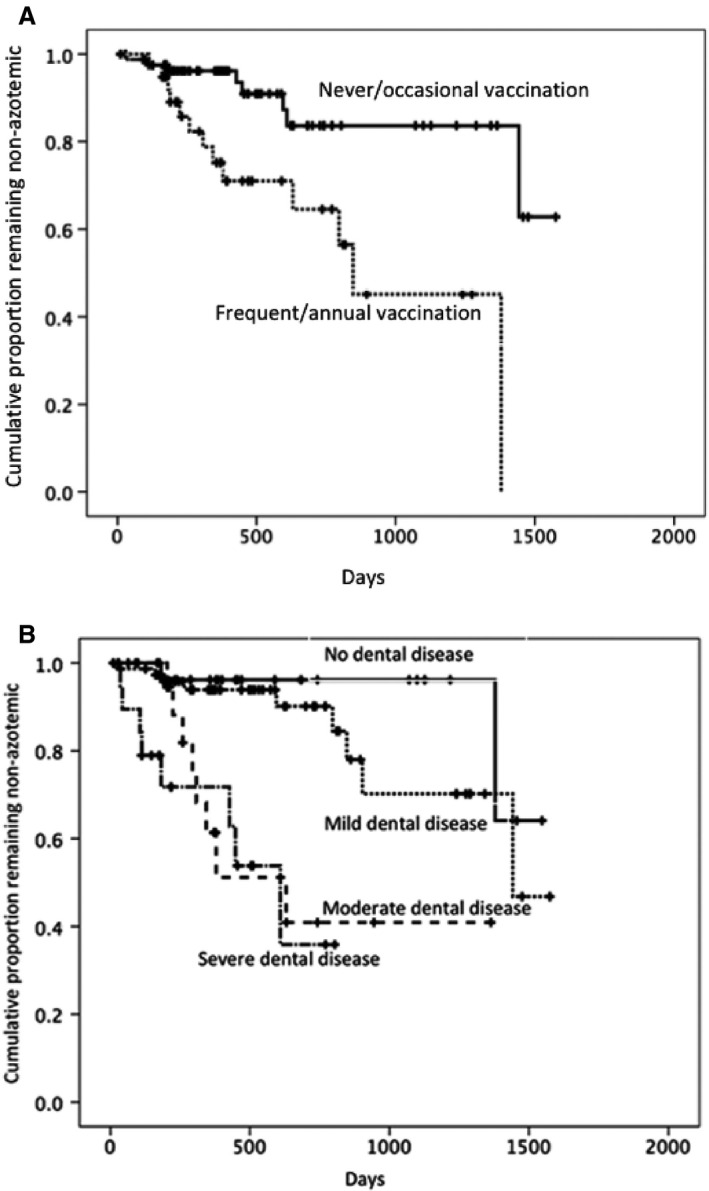
Kaplan–Meier curve of the risk of developing azotemia. (**A**) Vaccination status. (**B**) Dental disease category.

The following variables were significant in the univariable Cox regression analysis of risk factors for development of azotemia: age (*P* = .018), frequent/annual vaccination (*P* = .003), moderate dental disease (*P* = .003), and severe dental disease (*P* = .001). Unknown or missing data categories for variables were not significant in the univariable analysis Results of the univariable Cox regression analysis including hazard ratio and 95% confidence intervals are presented in Table 3. In the final multivariable model, the following variables remained independent risk factors for development of azotemia: frequent/annual vaccination (*P* = .003), moderate dental disease (*P* = .008), and severe dental disease (*P* = .001). Examination of the correlations matrix identified no variables to be highly correlated and no significant interactions among variables was identified. Results of the final multivariable Cox regression model are presented in Table 4. Kaplan–Meier curves for variables found to be significant in the final multivariable Cox regression model (vaccination status and dental disease category) are presented in Fig 1. A significant difference was identified between cats receiving frequent/annual vaccinations and cats that received no/occasional vaccinations when Kaplan–Meier curves were analyzed using the log rank test (*P* = .016). A significant difference between different categories of dental disease was identified when Kaplan–Meier curves were analyzed using the log rank test (*P* < .001).

**Table 3:** Results of the univariable Cox regression analysis of risk factors for development of azotemia in cats

Variable	Hazard Ratio	95% CI For Hazard Ratio		*P* Value
		Lower	Upper	
Age (years)	1.19	1.03	1.38	**.018**
Breed	0.74	0.27	1.98	.543
Sex	1.26	0.57	32.80	.567
Diet fed				
Approximately 50%:50%	0.60	0.16	2.22	.440
Predominantly wet food	1.30	0.55	3.05	.547
Urinary diet fed	0.51	0.07	3.84	.516
Senior diet fed	0.85	0.36	2.01	.705
Environment	1.49	0.44	5.02	.519
Lifestyle				
Approximately 50%:50%	1.21	0.39	3.71	.743
Predominantly outdoor	2.27	0.91	5.66	.077
Smoke exposure	1.57	0.70	3.52	.273
Frequent/annual vaccination status	3.92	1.60	9.61	**.003**
Dental disease previously diagnosed or treated	1.92	0.81	4.60	.140
Dental disease category				
Mild	2.35	0.50	10.97	.279
Moderate	11.02	2.20	55.09	**.003**
Severe	16.76	3.29	85.52	**.001**

*P* values for significant variables (*P* < .05) are highlighted in bold font. Reference categories for variables are presented in bold font in Table 1.

**Table 4:** Results of the final multivariable Cox regression model for risk factors for development of azotemia in cats

Variable	Β	SE	Hazard Ratio	95% CI for Hazard Ratio		*P* Value
				Lower	Upper	
Vaccination status						.008
Frequent/ annual vaccination	1.74	0.58	5.68	1.83	17.64	.003
Dental disease category						<.001
Mild	0.77	0.91	2.15	0.36	12.77	.398
Moderate	2.63	0.98	13.83	2.01	94.99	.008
Severe	3.57	1.07	35.35	4.31	289.73	.001

The reference category for vaccination status was never/occasional vaccination and for dental disease was no dental disease. B is the beta coefficient of the variable.

### Geospatial information system (GIS) analysis of geographical data

**Figure 2:**
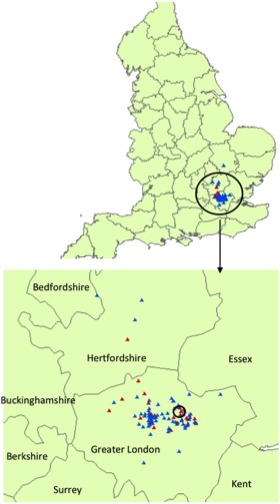
Geographical location of cats included in the study. Map of the geographical location of cats remaining nonazotemic (blue triangles) and cats developing azotemia (red triangles). Enlarged is a map of greater London and the counties surrounding London where the majority of cats lived. A single cluster of azotemic cats was detected (circled on enlarged map), however, this was not significant (*P* = .341).

Figure 2 presents cats developing azotemia and cats remaining nonazotemic projected onto a map of England. Results of the spatial scan analysis identified a single cluster of azotemic cats. However, this was not a statistically significant spatial cluster (*P* = .341).

## Discussion

Our study identified frequent or annual vaccination and moderate and severe dental disease as risk factors for development of azotemic CKD in geriatric cats.

Research in human patients has indicated that oral infections such as periodontal disease are not only local diseases affecting oral tissues but have systemic effects.^19^ In addition, periodontal disease has been identified as a risk factor for CKD in human patients,^20^, ^21^ and systematic review supports an association of periodontal disease and CKD.^22^ A retrospective case–control study found dental disease to be a risk factor for CKD in dogs but not cats.^5^ However, a more recent retrospective study identified prior diagnosis of periodontal disease to be a risk factor for CKD in cats although it was not possible to evaluate if dental disease preceded CKD development.^4^ The present study also suggests dental disease may be a risk factor for cats. Periodontal disease grade in dogs predicted histopathological changes in renal glomeruli and interstitium.^23^ A different study also found correlations between renal pathology and periodontal disease in dogs,^24^ suggesting that periodontal disease may contribute to development of chronic disease as a result of persistent low‐grade insults. Mechanisms by which periodontal disease may cause kidney injury include host factors, such as production of inflammatory cytokines or endotoxemia, and immune responses to bacteria. Chronic inflammatory responses secondary to periodontal disease may play a role in development of CKD in human patients. C‐reactive protein, an inflammatory biomarker, is increased in human patients with CKD.^25^, ^26^ Leukocytosis and hypoalbuminaemia, considered to be markers of systemic inflammation, also predicted future risk for CKD in human subjects.^27^ A limitation of our study is that confounding factors influencing dental disease as a predictor of development of azotemia cannot be excluded. Several infectious diseases are associated with gingivitis in cats and may play a role in development of kidney disease. Another potential confounder may be the effect of management of dental disease in cats including the use of antibiotics or nonsteroidal anti‐inflammatory drugs (NSAIDs) or a history of general anesthesia required for a dental procedure. Previous history of dental disease management was included in the analysis and not found to be significant in the final model. This finding suggests that factors related to dental disease itself, rather than its management, are important in development of azotemia in cats. Calculus and gingivitis index were assigned by a number of different clinicians, which also is a limitation of our study because neither intra‐ nor inter‐observer variability was assessed. In a proportion of cases (approximately 40%) the dental categorization system could not be applied and the clinician examining the cat had recorded the dental disease as mild, moderate, or severe or the category was defined based on the description of oral examination findings, therefore, we cannot be certain in these cases that the objective criteria were applied. Further studies are required to confirm these findings and also to explore the relationships among inflammatory biomarkers, dental disease, and CKD in cats. Furthermore, intervention studies also may be useful in evaluating if effective and early management of dental disease or preventative care programs are associated with a decrease in the incidence or progression of CKD. The effect of periodontal treatment in human patients on renal function remains uncertain.^22^ The severity of dental disease in our study may not be representative of the population of cats in the United Kingdom presented to veterinary practices in general because some of the cats included were presented to a charity hospital providing veterinary care for owners who are unable to afford regular veterinary fees, thus leading to a lack of early or preventative dental treatment.

The vaccinal viruses feline herpesvirus 1, calicivirus, and panleukopenia are cultured using CRFK cells. During manufacture of vaccines, CRFK proteins may become incorporated into vaccines to be administered to cats. Once vaccines are administered, cats may become exposed to CRFK antigens and develop an immune response. The CRFK cells are related to endogenous renal tissue and therefore it is possible cats may produce autoantibodies. In addition, immune complexes themselves may be damaging to the kidney although this generally would be associated with glomerulonephritis rather than tubulointerstitial nephritis which is the more common histopathological lesion in CKD in cats. Previous studies have suggested associations between kidney injury and vaccination in cats.^14^, ^15^, ^16^ Vaccination plays an important role in preventative feline medicine and its use has led to a decline in the prevalence of many infectious diseases. Nevertheless, results of the present study suggest vaccination may be a risk factor for development of CKD in cats, which raises some concern regarding frequent vaccination. In the present study, approximately 26% of all cats were frequently vaccinated. This is lower than a previous UK study that reported that 69% of cats belonging to owners completing a web‐based questionnaire were vaccinated.^28^ The reason for this disparity is unclear but may be attributable to differences in attitudes of the owners of cats included in each study (owners completing a questionnaire online regarding feline health may be more inclined to vaccinate their cats). In addition, vaccination was not offered at 1 of the clinics during part of the study period, and therefore cats were required to be taken to another clinic to receive vaccination. Further studies exploring the pathophysiological mechanism of renal injury associated with vaccination, perhaps by evaluating antirenal tissue antibodies, might advance our understanding of the relationship between frequency of vaccination and risk of developing azotemic CKD.

Results of our multivariate analysis regarding lifestyle variables are in agreement with that of a case–control study exploring risk factors for kidney disease in cats.^6^ Both studies found lifestyle variables such as environment and indoor/outdoor status not to be significantly associated with CKD in cats. Region of the United States in which a cat lived was reported to be a risk factor for CKD.^4^ To further evaluate the role of environment in development of CKD, GIS software was used. A single cluster of azotemic cats was detected, but, the cluster was not significant indicating a random distribution of disease. Analysis of geographical information supports the finding from the questionnaire data that the environment lived in (urban or semirural/rural) is not a risk factor for CKD in cats. However, an important consideration in interpreting these results is that most of the cats in the present study lived in an urban environment and were from a relatively small area within the United Kingdom (London and the surrounding area).

Glomerular filtration rate (GFR) decreases with age in human patients and this decrease has been identified in both cross‐sectional^29^ and longitudinal studies.^30^ Descriptive studies indicate that the prevalence of CKD increases with age in cats.^2^, ^31^ Results of studies exploring the relationship between GFR and age in cats have been conflicting with some studies reporting no correlation^32^ and others reporting an effect of age.^33^ A decrease in renal function with age also has been proposed to be a survival‐driven adaptive process in cats aimed at preserving life.^34^ In the present study, age was not found to be an independent predictor for development of azotemia in cats. However, the data are limited in that many cat owners were unable to provide an accurate age but rather an estimate only. Furthermore, all cats included in our study were geriatric (>9 years old) and a more varied age population may have resulted in different findings.

High protein intake is a dietary factor associated with progression of CKD in dogs^35^ and humans.^36^ Studies of cats with surgically induced models of kidney disease did not identify effects of dietary protein content on renal function.^37^, ^38^ Senior diets for cats typically are lower in protein content. In our study, there was no difference in the rate of development of azotemia in cats fed standard adult diets compared to senior diets suggesting no effect of protein content on the development of azotemia. A limitation is that information regarding specific diets fed to cats was highly variable among cats and the questionnaire data analyzed in the study did not include lifetime dietary information. Therefore, detailed analysis of protein and phosphorous content could not be performed. In addition, type of diet fed (wet versus dry) also was not a significant risk factor for development of CKD in the present study, which is in accordance with results from a previous study.^4^

Cigarette smoking has been identified in human patients as a risk factor for CKD.^20^, ^21^, ^39^, ^40^, ^41^ Healthy rats exposed to passive smoke over a 4‐month period showed no difference in histopathology of the kidney compared to control rats.^42^ However, a longer term study of rats exposed to passive smoke at a concentration similar to human passive smoking, found significant histopathological evidence of glomerulosclerosis and tubulointerstitial fibrosis compared to control rats.^43^ Results of the present study suggest that passive smoking is not an important risk factor for development of azotemia in cats. However, level of exposure could not be evaluated and dose‐dependent studies may be required to determine any effect.

The mean incidence of development of azotemic CKD within 12 months in our study was 11%. An earlier study using data from our group reported that 30.5% of cats developed azotemia within 12 months.^3^ This discrepancy likely is related to age differences between the 2 populations with the cats developing azotemic CKD in the earlier study^3^ having a median age of 14.6 years at baseline compared to the cats developing azotemic CKD in the present study that had a median age of 13.4 years.

Several possible risk factors for CKD in cats are of interest, but they were not explored in our study. Infection with FIV is reported to be a risk factor for CKD in cats,^7^, ^44^ but the association is reported only in younger and not older cats.^7^ Histopathological examination of renal tissue from cats infected with FIV shows tubulointerstitial lesions, glomerulosclerosis, amyloid deposits, and high viral antigen load in tubular epithelial cells in a large proportion of cats.^44^, ^45^ Cats included in the present study were not routinely screened for FIV status. It would be interesting to evaluate this risk factor further. Nevertheless, the importance of FIV infection in the older feline population is unclear. Genetic factors may contribute to development of CKD but remain to be explored in cats. With increasing advances in the field of feline genetics it is likely further evidence for the role of genetic factors in development of CKD will be identified. Prior exposure to renal toxins (eg, NSAIDs, antineoplastic drugs, mycotoxins, heavy metals, Easter lilies) is an important risk factor to consider but was not evaluated in the present study because collection of data regarding toxin exposure by means of a questionnaire would have been difficult. Many toxins are known to cause acute kidney injury at high doses, but the role of low‐dose cumulative exposure in development of CKD in cats remains unknown.

Several limitations of our study warrant comment. The first is use of plasma creatinine concentration (ie, azotemia) as a marker of renal function. The exponential relationship between plasma creatinine concentration and GFR means that creatinine is not a sensitive marker for detecting early decrease in renal function. Furthermore, decreased muscle mass will result in decreased endogenous production of creatinine. Prospective longitudinal studies evaluating predictors and risk factors for decreasing GFR in cats may have been more optimal. Obtaining vaccination history from owners may be an inaccurate method of collecting information because owners may be unable to provide an accurate vaccination history. Social desirability bias also may result in owners responding to the question in a way that is inaccurate because they feel this would be viewed more favorably. This limitation could have been avoided by obtaining vaccination history from practice records. Doing so would have been extremely difficult because many owners presented their cats to several clinics. Furthermore, computer records at both first opinion practices only were available for a limited number of years because of changes in systems. Support for the use of a questionnaire to collect data regarding vaccination history comes from a previous study exploring owner attitudes to vaccination in cats.^28^ Our study used a web‐based questionnaire completed by owners and was found to be a satisfactory method to collect data.

Early identification of cats with CKD is desirable because interventional treatment can be implemented which may attenuate progression of disease and prevent secondary metabolic complications. Population‐based screening may improve detection of CKD, it is expensive, however, and it may be difficult to convince many owners that testing is necessary, particularly if their pet appears clinically healthy or is showing only very subtle clinical signs. Therefore, targeted screening is likely to be a more useful approach. Evidence from human medicine suggests that population‐based screening is not cost effective and that targeted screening is more valuable.^46^ Targeted screening has proved to be successful in human patients with establishment of the National Kidney Foundation's Kidney Early Evaluation Program (KEEP). Targeted screening requires identification of risk factors to establish at‐risk cats within the population. Future work investigating risk factors identified in the present study may address development of risk scores for CKD in cats. A useful tool for clinical practice could be developed based on weighted scores of multiple factors entered by practitioners to define an individual cat's risk of developing azotemia. Cats identified to have a high risk would be recommended to be screened for CKD. In human patients, the development of categorization systems based on certain risk factors such as age, sex, hypertension, diabetes mellitus, cardiovascular disease, and anemia have proved to be helpful in identifying patients in the population at risk of developing CKD.^1^, ^47^

Results of our study suggest there is no single risk factor, exposure, or predictor that can explain development of CKD in cats and therefore cumulative effects of multiple risk factors and interactive factors should be considered. Cumulative exposure to risk factors in certain possibly genetically predisposed cats may contribute to a decrease in renal function. The rate of decrease also may be accelerated by additional risk factors once CKD has developed. Host factors may play a role as may exogenous factors such as frequent vaccination and the presence of dental disease. Epidemiological studies, as conducted in our study, do not necessarily imply causality, but simply suggest associations. Prevention of decreased renal function may involve minimization of lifelong exposure to risk factors. Doing so potentially includes maintenance of good oral health and avoiding excessive vaccination of cats.

## Footnotes

1 Redlands, CA

2 SPSS version 17.0
